# Drug-eluting balloon versus bare-mental stent and drug-eluting stent for de novo coronary artery disease: A systematic review and meta-analysis of 14 randomized controlled trials

**DOI:** 10.1371/journal.pone.0176365

**Published:** 2017-04-26

**Authors:** Kongyong Cui, Shuzheng Lyu, Xiantao Song, Fei Yuan, Feng Xu, Min Zhang, Wei Wang, Dongfeng Zhang, Jing Dai

**Affiliations:** Department of Cardiology, Beijing Anzhen Hospital, Capital Medical University and Beijing Institute of Heart, Lung and Blood Vessel Diseases, Beijing, China; University of Bologna, ITALY

## Abstract

**Background:**

Drug-eluting balloon (DEB) has become an alternative option to drug-eluting stent (DES) for the treatment of in-stent restenosis (ISR). However, the effect of drug-eluting balloon with regular bare-mental stent (BMS) in *de novo* coronary artery disease (CAD) is unclear. This meta-analysis aimed to evaluate the efficacy of DEB with regular BMS compared to BMS or DES in *de novo* CAD.

**Methods:**

Randomized controlled trials (RCTs) assessing the efficacy of DEB+BMS in comparison with BMS or DES were obtained by searching the PubMed, EMBASE, and Cochrane Library databases through January 2016. Primary endpoints were major adverse cardiac events (MACEs) and late lumen loss (LLL). Secondary endpoints included death, myocardial infarction (MI), target lesion revascularization (TLR), stent thrombosis (ST), binary restenosis, and minimum lumen diameter (MLD). Dichotomous and continuous data were presented as odds ratios (ORs) and mean differences (MDs) with 95% confidence intervals (CIs), respectively, and analyzed using a random-effects model.

**Results:**

A total of 14 RCTs involving 2281 patients were included in this meta-analysis. DEB+BMS showed significantly less MACEs (OR: 0.67, 95%CI 0.45 to 0.99, P = 0.04) and reduced LLL (MD: -0.30 mm, 95%CI: -0.48 mm to -0.11 mm, P = 0.001) compared with BMS. Meanwhile, treatment with DEB+BMS had disadvantages over DES in terms of MACEs (OR: 1.94, 95%CI 1.24 to 3.05, P = 0.004), LLL (MD: 0.20 mm, 95%CI: 0.07 mm to 0.33 mm, P = 0.003), TLR (OR: 2.53, 95% CI 1.36 to 4.72, P = 0.003), and MLD (MD: -0.25 mm, 95%CI: -0.42 mm to -0.09 mm, P = 0.003).

**Conclusions:**

This limited evidence demonstrated that treatment with DEB+BMS appears to be effective in *de novo* CAD. In addition, DEB+ BMS clearly showed superiority to BMS, but is inferior to DES in the treatment of patients with *de novo* CAD. Hence, DES (especially new generation DES) should be recommended for patients with *de novo* CAD.

## Introduction

First-generation drug-eluting stents (DESs) reduce restenosis after percutaneous coronary intervention (PCI) by preventing vessel wall recoil and late negative remodeling, as well as restraining neointimal hyperplasia [1,2]. However, they are associated with many potential serious complications such as late stent thrombosis, non-homogenous drug delivery, and delayed vascularization, which makes bare-mental stent (BMS) preferred with shorter dual antiplatelet therapy in patients at high bleeding risk [3,4]. Recently, network meta-analyses has demonstrated new generation DES was associated with significantly lower rates of stent thrombosis in comparison to BMS [5,6]. Furthermore, randomized controlled trials (RCTs) with new generation DES have shown lower rate of stent thrombosis and similar risk of bleeding compared with BMS in patients with contraindications to first-generation DES [7,8]. Obviously, new generation DES might be an appropriate choice for the treatment of *de novo* coronary disease among patients at high risk of bleeding or thrombosis.

Local drug delivery by drug-eluting balloon (DEB) has emerged as an effective and safe treatment option for in-stent restenosis (ISR) in both BMS [9] and DES [10,11], delivering active drugs homogeneously to inhibit neointimal hyperplasia without remaining in the arteries permanently. To date, DEB is considered an important method for treating BMS-ISR and DES-ISR in the updated European Society of Cardiology guidelines with a class I recommendation (level of evidence A) [12]. Furthermore, DEB can also deliver drugs to *de novo* coronary lesions. Indeed, it is usually used in combination with BMS, especially for lesions without local flow-limiting vessel dissections and high-grade elastic recoil. Although previous meta-analyses demonstrated that DEB+BMS was not superior to BMS, while DEB with/without BMS tended to be inferior to DES without statistical differences, the effect of DEB with regular BMS compared with BMS or DES in *de novo* coronary artery disease (CAD) remains unclear and the topic is challenging till now [13,14]. Firstly, the studies were restricted to small sample size [13,14]. In fact, DEB was not widespread for *de novo* coronary lesions due to lack of recommendation in contemporary guidelines and the development of new generation DES and bioresorbable stent. Secondly, studies that applied both DEB alone and DEB with regular BMS were included in previous meta-analyses, which may cause severe heterogeneity [13,14]. Thirdly, first-generation DES was used in most of the studies, whereas DEB should compare with current standard of new generation DES with the development of stent design. Recently, several RCTs using DEB+BMS to treat *de novo* lesions comparatively with BMS or DES treatments were reported. Importantly, new generation DES was widely adapted in these studies. We performed an updated meta-analysis of all currently available RCTs to evaluate DEB+BMS efficacy in the treatment of *de novo* CAD.

## Methods

This study was carried out in compliance with the Preferred Reporting Items for Systematic Reviews and Meta-Analyses (PRISMA) statement [15].

### Search strategy

RCTs investigating the efficacy of DEB+BMS comparatively to BMS or DES in *de novo* CAD were included. Pertinent articles were identified through a comprehensive search of electronic databases, including PubMed, EMBASE, and Cochrane Library through January 2016. Only studies published in English and limited to human subjects were taken into account. The following medical subject headings and search terms were used: “drug eluting balloon”, “drug coated balloon”, “paclitaxel coated balloon”, and “paclitaxel eluting balloon”. The references in the identified articles and relevant reviews were screened to include other potentially suitable trials. The authors of the original studies were not contacted for additional information.

### Study selection

Studies were included if they: (1) were RCTs associated with *de novo* CAD; (2) were published in English and limited to human subjects; (3) compared DEB+BMS to BMS or DES; (4) had subjects followed for at least 6 months; (5) assessed the endpoints of interest. At first, the selection was conducted by screening titles or abstracts; then, full-text reviews were performed. When several reports overlapped, only the largest and latest one was selected. All articles were reviewed by two independent investigators (WW and JD) to determine whether they met the inclusion criteria, and any disagreement was resolved by consensus.

### Data extraction

The following data were extracted using a standardized form for each eligible article: study characteristics, patient characteristics, and angiographic and clinical outcomes. Primary endpoints were major adverse cardiac events (MACEs) and late lumen loss (LLL). MACEs were defined as a composite of death, MI, and TLR, while the definition of LLL was the difference between the postprocedural and follow-up minimum lumen diameter (MLD) in the same segment. The most similar endpoint was chosen in case one endpoint was not reported. Definitions of MACEs in the individual studies are shown in Table 1. Secondary endpoints included death, myocardial infarction (MI), target lesion revascularization (TLR, defined as any repeat revascularization in the treated segment), stent thrombosis (ST, classified according to the Academic Research Consortium definition [16]), binary restenosis (BR, defined as >50% of diameter stenosis), and MLD. Data extraction was performed by two independent investigators (FX and MZ), and differences in assessments were resolved by discussing with a third investigator (FY).

**Table 1:** Study and population characteristics.

Primary author, year published	Enrollment period and country	Patient characteristics	Comparison	Total no. patients	DEB±BMS(n)	BMS(n)	DES(n)	Clopidogrel (mts)	Angiographic f/u(mts)	Clinical f/u(mts)	MACE
**Ali 2011**[18]	2007.5–2009.1;multicenter;Malaysia and Thailand	SA or UA with diabetes mellitus; single de novo stenosis	SeQuent Please DEB+BMS vs. Taxus Liberté PES	84	45	NA	39	DEB+BMS 3,DES 6	9	9	Death, MI, TLR, and ST
**Belkacemi 2011**[19,33]	2007.11–2009.12;multicenter;Netherlands,Belgium, and Germany	SA or UA; bifurcation	DIOR-I DEB+BMS vs. BMS vs. Taxus Liberte PES	117	40	37	40	DEB+BMS 3,DES 12	6	18	Death, MI, TLR
**Belkacemi 2012**[20]	2009.2–2010.11;multicenter;Netherlands,Italy	STEMI; a single culprit lesion in the target vessel	DIOR-II DEB+ BMS vs. BMS vs. Taxus Liberte PES	150	50	51	49	12	6	6	Death, MI, TVR
**Besic 2015**[21]	2011.2–2013.6;single-center;Croatia	NSTEMI or UA; de novo coronary lesions	Elutax/SeQuent Please DEB+BMS vs. BMS	85	41	44	NA	12	6	6	TLR, ST, and ACS
**Burzotta 2015**[22]	2 years; NA; Italy	SA, non-diabetic; de novo, non-complex lesions	IN.PACT Falcon DEB+ BMS vs. BMS	30	20	10	NA	3	6	12	Death, MI, TLR
**Camaro 2015**[23]	NA; multicenteer; Netherlands	STEMI	paclitaxel-coated DEB+EPC vs. EPC	130	NA	NA	NA	12	9	12	Death, MI, TVR
**Hamm 2009**[24]	2007.7–2008.9;multicenter;Germany and Belgium	SA or UA; single de novo stenosis	Coroflex DEBlue DEB+BMS vs. Cypher SES	637	312	NA	325	12	9	9	Death, MI, and any revascularization
**Liistro 2013**[25]	2009.1–2009.10;single-center;Italy	stable angina; single de novo stenosis	Elutax DEB+ BMS vs. Xience V EES	125	59	NA	66	DEB+BMS 3,DES 12	9	9	Death, MI, TVR
**Lopez 2014**[26]	2010.1–2012.1;multicenter;Spanish	SA or UA; bifurcation	SeQuent Please DEB+ BMS vs. Xience V EES	108	52	NA	56	DEB+BMS 3,DES 12	9	24	Death, MI, TLR
**Poerner 2014**[27]	2009.6–2011.2;single-center;Germany	suspected or previously documented CAD; native coronary lesions suitable for stent placement	SeQuent Please DEB+BMS vs. Xience V EES	90*	51	NA	48	12	6	6	Death, MI, revascularization
**Seeger 2016**[28]	2009.2–2010.2;multicenter;NA	SA or UA; single de novo stenosis	Sequent Please DEB+EPC vs. EPC	120	62	58	NA	3	6	60	Death, MI, TLR
**Touchard 2015**[29]	NA; multicenter; Spain	STEMI	paclitaxel-coated DEB+BMS vs. BMS	223	111	112	NA	NA	9	12	Death, MI, acute CAD, hemorrhagy and/or ST
**Yoon 2015**[30]	NA	SA, UA or NSTEMI	Sequent Please DEB+ BMS vs. Resolute Integrity ZES	180	90	NA	90	NA	9	9	NA
**Zurakowski 2015**[31]	2011–2012;multicenter;Poland	SA or UA; single de novo stenosis	SeQuent Please DEB+ BMS vs. Coroflex Please PES	202	102	NA	100	12	9	9	Death, MI, TVR

no., number of; f/u, follow up; mts, months; NA, not applicable; DEB, drug-eluting balloon; BMS, bare-metal stent; EPC, endothelial progenitor cell capturing; DES, drug-eluting stent; PES, paclitaxel-eluting stent; SES, sirolimus-eluting stent; EES, everolimus-eluting stent; ZES, zotarolimus-eluting stent; MACE, major adverse cardiac events; TLR, target lesion revascularization; TVR, target vessel revascularization; ST, stent thrombosis; CAD, coronary artery disease; SA, stable angina; UA, unstable angina; MI, myocardial infarction; STEMI, ST-segment elevation myocardial infarction; NSTEMI, non ST-segment elevation myocardial infarction; ACS, acute coronary syndrome;

* 9 patients with >1 suitable leasion were sequentially included in both device groups

### Quality assessment

The quality of eligible articles was assessed by evaluating the following methodological criteria recommended by the Cochrane Collaboration: sequence generation, concealment of allocation, blinding, incomplete outcome data, selective outcome reporting, and other sources of bias [17].

### Statistical analysis

All statistical analyses were carried out with Review Manager 5.1 (Cochrane Center, Denmark). Dichotomous data were presented as odds ratios (ORs) with 95% confidence intervals (CIs): these included MACEs, death, MI, TLR, ST and BR. A quantitative analysis was performed to estimate the mean differences (MDs) for continuous variables with 95% CI. Potential heterogeneity among trials was assessed with the I^2^ statistical test. An I^2^ value exceeding 50% was defined as statistical heterogeneity. For ORs, the Dersimonian and Lair random-effects model was used, while the overall mean difference was constructed with the Inverse Variance random-effects model.

Sensitivity analyses were performed to demonstrate the robustness of results by removing studies according to the conditions described below. For DEB+BMS vs. BMS: (1) excluding endothelial progenitor cell capturing (EPC) stent; (2) excluding ST-segment elevation myocardial infarction (STEMI); (3) excluding bifurcations. In the case of DEB+BMS vs. DES: (1) excluding STEMI; (2) excluding bifurcations; (3) SeQuent Please exclusively; (4) paclitaxel-eluting stent (PES) exclusively; (5) everolimus-eluting stent (EES) exclusively; (6) published in full text exclusively. Sensitivity analyses were also performed by omitting one study in each turn. Two-sided P values <0.05 were considered statistically significant.

## Results

### Eligible studies

**Fig 1:**
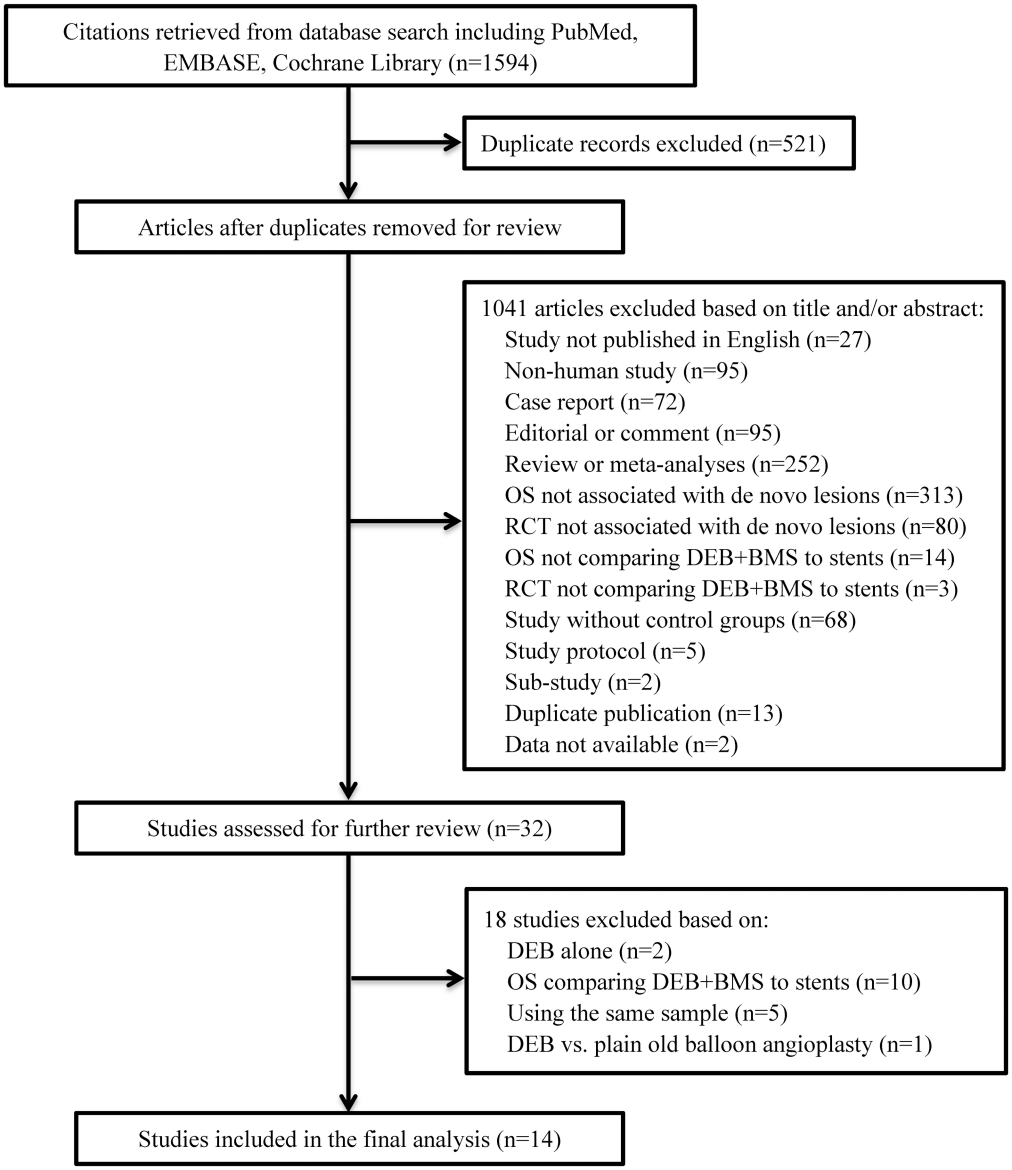
Process for study selection OS, observational study.

After a comprehensive review of the retrieved articles, 1594 potentially related reports were identified in the initial analysis. A total of 521 articles were removed due to duplication and 1041 excluded after initial screening based on title and/or abstract. A total of 32 articles were selected for complete review. Finally, 14 RCTs [18,19,20,21,22,23,24,25,26,27,28,29,30,31] involving 2281 patients were included in the current meta-analysis. The study selection process is summarized in Fig 1. In the OCTOPUS study, 24 month clinical outcomes were reported as an abstract [32], with data not yet available; therefore the related study with six-month angiographic and clinical follow up data was enrolled [27].

Study and population characteristics are presented in Table 1. Of the 14 RCTs, 7 compared DEB+BMS and DES [18,24,25,26,27,30,31]; meanwhile, 5 trials compared DEB+BMS and BMS [21,22,23,28,29], and the remaining 2 were 3-arm trials comparing DEB+BMS, BMS, and DES [19,20]. The DEBs used in original trials were SeQuent Please [18,26,27,28,30,31], Dior [19,20], IN.PACT Falcon [22], and other paclitaxel-eluting balloons. One study [21] used two DEB types, including SeQuent Please and Elutax. The devices used in control arms were BMS (7 arms) and DES (9 arms); regarding the type of DES, PES (4 arms), EES (3 arms), zotarolimus-eluting stent (ZES) (1 arm) and sirolimus-eluting stent (SES) (1 arm) were included. Overall, 3 trials enrolled patients with STEMI [20,23,29]; in the remaining 11 trials, patients with stable angina, unstable angina or non-ST segment elevation myocardial infarction were enrolled. Concerning lesion subsets, bifurcations [19,26] and simple *de novo* lesions [18,20,21,22,23,24,25,27,28,29,30,31] were all included in this meta-analysis. Clinical follow-up period ranged from 6 to 60 months; the duration of angiographic follow-up varied between 6 and 9 months. Quality assessment results are detailed in Table 2.

**Table 2:** Assessment of randomized controlled trials.

Primary author, year published	Sequence generation	Concealment of allocation	blinding of participants, personnel and outcome assessors	Incomplete outcome data addressed	Free of selective reporting	Free of other bias
**Ali 2011**[18]	NA	NA	Yes	Yes	Yes	Yes
**Belkacemi 2011**[19,33]	Yes	Yes	Yes	Yes	Yes	Yes
**Belkacemi 2012**[20]	Yes	Yes	Yes	Yes	Yes	Yes
**Besic 2015**[21]	NA	NA	NA	Yes	Yes	Yes
**Burzotta 2015**[22]	NA	NA	Yes	Yes	Yes	Yes
**Camaro 2015**[23]	NA	NA	NA	NA	NA	NA
**Hamm 2009**[24,34]	NA	NA	Yes	Yes	Yes	Yes
**Liistro 2013**[25]	Yes	Yes	Yes	Yes	Yes	Yes
**Lopez 2014**[26]	Yes	NA	Yes	Yes	Yes	Yes
**Poerner 2014**[27]	NA	NA	Yes	Yes	Yes	Yes
**Seeger 2016**[28]	Yes	NA	Yes	Yes	Yes	Yes
**Touchard 2015**[29]	NA	NA	NA	NA	NA	NA
**Yoon 2015**[30]	NA	NA	NA	NA	NA	NA
**Zurakowski 2015**[31]	Yes	NA	Yes	Yes	Yes	Yes

NA, not applicable;

### Primary endpoints

#### Major adverse cardiac events

**Fig 2:**
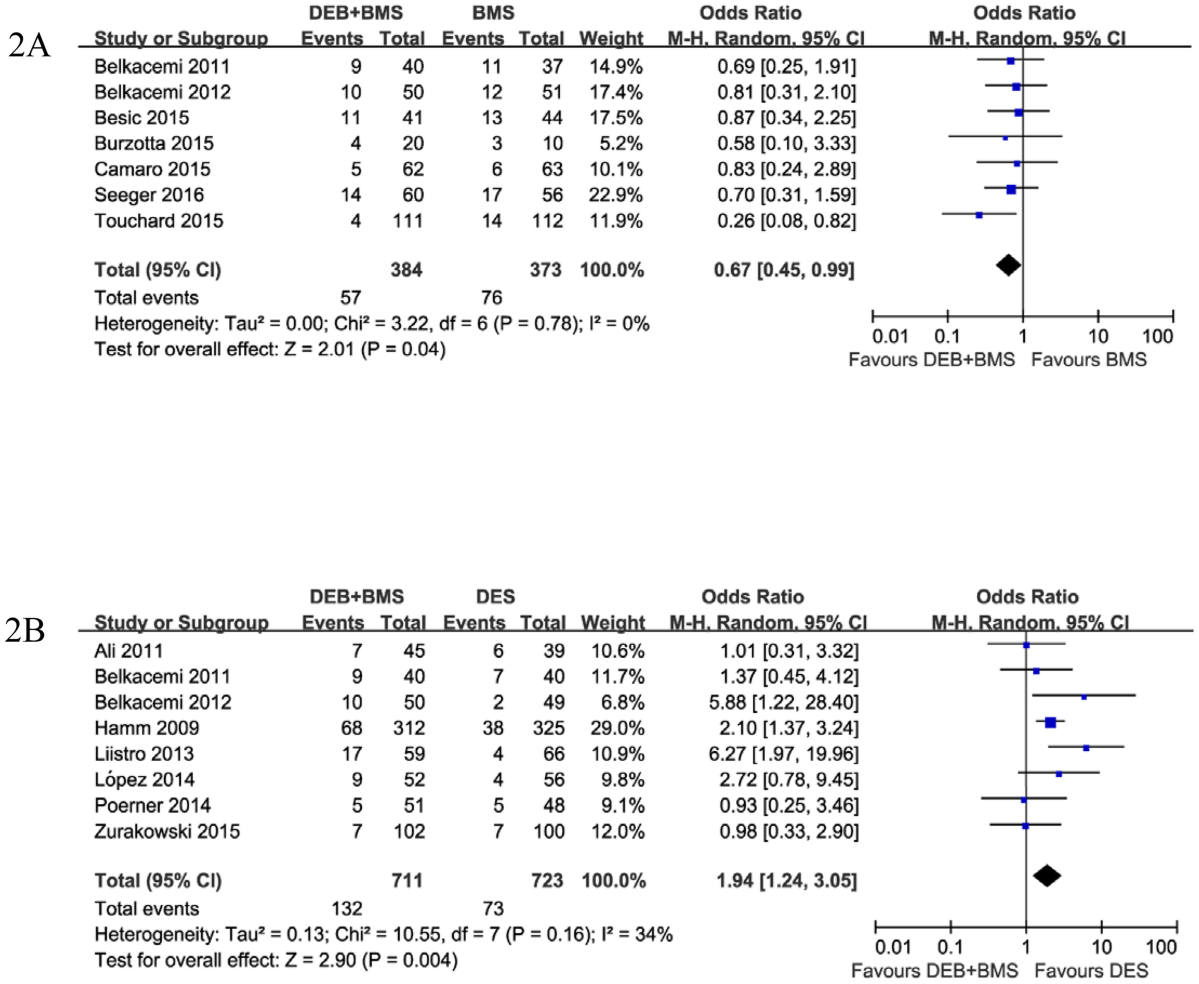
Forest plot of major adverse cardiac events comparing DEB+BMS with (A) BMS and (B) DES.

Overall, MACEs were reported in 13 studies (28 arms). MACEs occurred in 170 (16.9%), 76 (20.4%) and 73 (10.1%) patients in the DEB+BMS, BMS and DES groups, respectively. The pooled OR for MACEs is shown in Fig 2A and 2B. Compared with the BMS group, treatment with DEB+BMS was associated with a lower risk of MACEs (OR: 0.67, 95%CI 0.45 to 0.99, P = 0.04, I^2^ = 0%) (Fig 2A). In contrast, the risk of MACEs was significantly reduced in the DES group compared with DEB+BMS treated patients (OR: 1.94, 95%CI 1.24 to 3.05, P = 0.004, I^2^ = 34%) (Fig 2B).

#### Late lumen loss

**Fig 3:**
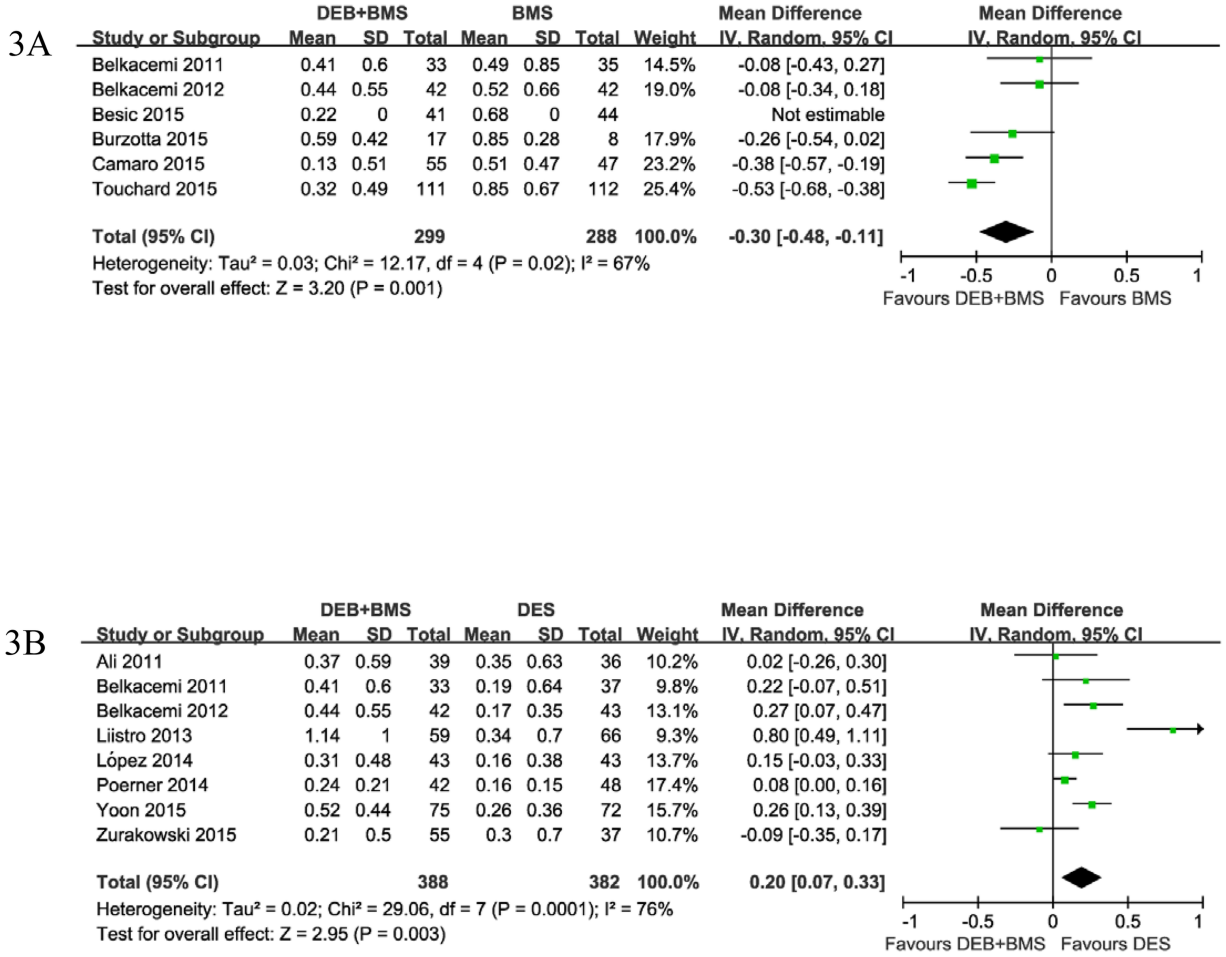
Forest plot of late lumen loss comparing DEB+BMS with (A) BMS and (B) DES.

As shown in Fig 3A and 3B, LLL data were available in 12 trials (26 arms). Compared with the BMS group, treatment with DEB+BMS was associated with reduced LLL (MD: -0.30 mm, 95%CI: -0.48 mm to -0.11 mm, P = 0.001, I^2^ = 67%) (Fig 3A); meanwhile, the DEB+BMS group was inferior to the DES group in terms of LLL (MD: 0.20 mm, 95%CI: 0.07 mm to 0.33 mm, P = 0.003, I^2^ = 76%) (Fig 3B).

### Secondary outcomes

#### Death

The rate of death was not significantly different for DEB+BMS vs. BMS (OR: 1.13, 95% CI 0.41 to 3.09, P = 0.81, I^2^ = 0%) and DEB+BMS vs. DES (OR: 2.14, 95% CI 0.51 to 9.00, P = 0.30, I^2^ = 0%), respectively (Table 3).

**Table 3:** Secondary endpoints.

Outcomes	Comparison	No. Trials	No. patients (or lesions)*	OR/MD (95% CI)	P value	I^2^, %
**Death**	DEB+BMS vs. BMS	5	211/198	1.13[0.41, 3.09]	0.81	0%
	DEB+BMS vs. DES	8	489/490	2.14[0.51, 9.00]	0.30	0%
**MI**	DEB+BMS vs. BMS	4	170/154	2.12[0.62, 7.26]	0.23	0%
	DEB+BMS vs. DES	8	489/490	1.19[0.47, 3.04]	0.72	0%
**TLR**	DEB+BMS vs. BMS	6	322/310	0.69[0.43, 1.10]	0.12	0%
	DEB+BMS vs. DES	8	699/715	2.53[1.36, 4.72]	0.003	38%
**ST**	DEB+BMS vs. BMS	5	302/300	2.06[0.42, 10.11]	0.37	0%
	DEB+BMS vs. DES	4	237/228	1.27[0.41, 3.98]	0.68	0%
**BR**	DEB+BMS vs. BMS	4	227/233	0.47[0.12, 1.84]	0.28	83%
	DEB+BMS vs. DES	7	322/323	2.26[1.01, 5.08]	0.05	58%
**MLD(mm)**	DEB+BMS vs. BMS	4	133/129	0.08[-0.11, 0.26]	0.42	0%
	DEB+BMS vs. DES	6	274/274	-0.25[-0.42, -0.09]	0.003	64%

MI, myocardial infarction; TLR, target lesion revascularization; ST, stent thrombosis; BR, binary restenosis; MLD, minimal lumen diameter; DEB, drug-eluting balloon; BMS, bare-mental stent; DES, drug-eluting stent; NO., number of; OR, odd ratio; MD, mean difference; CI, confidence internal;

*The numerals indicate the number of cases and controls, respectively.

#### Myocardial infarction

Overall, no statistically significant differences in MI were obtained between DEB+BMS and BMS (OR: 2.12, 95% CI 0.62 to 7.26, P = 0.23, I^2^ = 0%) or DEB+BMS and DES (OR: 1.19, 95% CI 0.47 to 3.04, P = 0.72, I^2^ = 0%) groups (Table 3).

#### Target lesion revascularization

A total of 12 trials (26 arms) reported target lesion revascularization. As shown in Table 3, pooled OR for TLR was similar between the DEB+BMS and BMS groups (OR: 0.69, 95% CI 0.43 to 1.10, P = 0.12, I^2^ = 0%). Nevertheless, DEB+BMS treatment was associated with a higher rate of TLR compared with the DES group (OR: 2.53, 95% CI 1.36 to 4.72, P = 0.003, I^2^ = 38%).

#### Stent thrombosis

The incidence of stent thrombosis was low in both groups. In the pooled estimate, the risk of stent thrombosis was similar between DEB+BMS and BMS groups (OR: 2.06, 95% CI 0.42 to 10.11, P = 0.37, I^2^ = 0%) or DEB+BMS and DES groups (OR: 1.27, 95% CI 0.41 to 3.98, P = 0.68, I^2^ = 0%) (Table 3).

#### Binary restenosis

Binary restenosis was reported in 9 trials (20 arms) by angiographic follow-up. As shown in Table 3, no significant differences were observed between DEB+BMS and BMS groups (OR: 0.47, 95%CI 0.12 to 1.84, P = 0.28, I^2^ = 83%). Treatment with DEB+BMS tended to be superior to DES in terms of binary restenosis (OR: 2.26, 95% CI 1.01 to 5.08, P = 0.05, I^2^ = 58%).

#### Minimum lumen diameter

Overall, 8 studies including 18 arms reported minimum lumen diameter data. The pooled MDs for MLD are shown in Table 3. MLD was similar between DEB+BMS and BMS groups (MD: 0.08 mm, 95% CI: -0.11 mm to 0.26 mm, P = 0.42, I^2^ = 0%). However, treatment with DEB+BMS was associated with reduced MLD compared with the DES group (MD: -0.25 mm, 95%CI: -0.42 mm to -0.09 mm, P = 0.003, I^2^ = 64%).

#### Sensitivity analysis

To assess the robustness of primary endpoint results, sensitivity analyses performed through removal of any single trial, which did not essentially affect the overall pooled estimate in the DEB+BMS vs. BMS and DEB+BMS vs. DES groups, respectively (data not shown).

Sensitivity analysis was also performed by evaluating the effects of various variables in the included trials (Table 4). Mostly similar results were obtained compared to the overall analysis. In the analysis comparing DEB+BMS to BMS, no statistical differences were found between the two groups after exclusion of patients with STEMI, although DEB+BMS was associated with a lower rate of MACEs (OR: 0.73, 95% CI 0.44 to 1.21, P = 0.23) and reduced LLL (MD: -0.19 mm, 95% CI: -0.41 mm to 0.03 mm, P = 0.09). On the other hand, comparing DEB+BMS to DES, analysis of trials using SeQuent Please showed that both groups were comparable in MACEs (OR: 0.98, 95% CI 0.49 to 1.94, P = 0.95) and LLL (MD: 0.10 mm, 95% CI: -0.04 to 0.23, P = 0.15). Furthermore, treatment with DEB+BMS was comparable to PES exclusively regarding MACEs (OR: 1.46, 95% CI 0.73 to 2.91, P = 0.29) and LLL (MD: 0.12 mm, 95% CI: -0.06 mm to 0.29 mm, P = 0.19).

**Table 4:** Sensitivity analyses.

Outcome	DEB+BMS vs. BMS			DEB+BMS vs. DES					
	Excluding EPC stent	Excluding STEMI	Excluding bifurcation	Excluding STEMI	Excluding bifurcation	SeQuent Please exclusively	PES exclusively	EES exclusively	Published in full text exclusively
**MACE**	0.64[0.39,1.03] P = 0.07	0.73[0.44,1.21] P = 0.23	0.66[0.43,1.02] P = 0.06	1.80[1.15,2.82] P = 0.01	1.96[1.09,3.55] P = 0.03	0.98[0.49,1.94] P = 0.95	1.46[0.73,2.91] P = 0.29	2.60[0.88,7.66] P = 0.08	2.04[0.99,4.20] P = 0.05
**LLL (mm)**	-0.26[-0.51,-0.01] P = 0.04	-0.19[-0.41,0.03] P = 0.09	-0.33[-0.52,-0.14] P = 0.0006	0.19[0.04,0.34] P = 0.01	0.21[0.04,0.38] P = 0.02	0.10[-0.04,0.23] P = 0.15	0.12[-0.06,0.29] P = 0.19	0.31[-0.01,0.63] P = 0.06	0.19[0.01,0.36] P = 0.04
**Death**	0.71[0.05,9.79] P = 0.80	1.40[0.48,4.05] P = 0.54	0.87[0.19,3.91] P = 0.86	2.14[0.51,9.00] P = 0.30	2.01[0.37,10.83] P = 0.42	2.01[0.37,10.83] P = 0.42	4.60[0.51,41.38] P = 0.17	4.90[0.23,104.7] P = 0.31	5.66[0.67,48.18] P = 0.11
**MI**	3.95[0.43,36.5] P = 0.23	1.77[0.46,6.81] P = 0.40	2.01[0.53,7.64] P = 0.30	1.02[0.38,2.74] P = 0.96	1.26[0.40,3.98] P = 0.69	1.20[0.31,4.57] P = 0.79	1.41[0.46,4.35] P = 0.54	0.80[0.15,4.37] P = 0.80	1.22[0.45.3.28] P = 0.70
**TLR**	0.73[0.43,1.24] P = 0.24	0.67[0.38,1.16] P = 0.15	0.69[0.41,1.16] P = 0.16	2.27[1.24,4.16] P = 0.008	2.59[1.15,5.86] P = 0.02	1.10[0.33,3.86] P = 0.87	1.87[0.43,8.09] P = 0.41	3.45[0.88,13.54] P = 0.08	2.83[0.83,9.61] P = 0.10
**ST**	3.81[0.61,23.69] P = 0.15	1.00[0.10,10.29] P = 1.00	2.06[0.42,10.11] P = 0.37	1.02[0.30,3.48] P = 0.98	1.54[0.46,5.23] P = 0.48	1.23[0.33,4.66] P = 0.76	1.27[0.41,3.98] P = 0.68	NA	1.54[0.46,5.23] P = 0.48
**BR**	0.47[0.12,1.84] P = 0.28	0.75[0.35,1.60] P = 0.46	0.38[0.05,2.66] P = 0.33	1.88[0.78,4.49] P = 0.16	2.20[0.63,7.66] P = 0.21	0.75[0.30,1.84] P = 0.53	1.52[0.59,3.88] P = 0.39	5.23[2.04,13.42] P = 0.0006	2.43[0.90,6.58] P = 0.08
**MLD (mm)**	0.08[-0.11,0.26] P = 0.42	0.02[-0.21,0.25] P = 0.84	0.07[-0.20,0.33] P = 0.63	-0.21[-0.39,-0.04] P = 0.02	-0.30[-0.55,-0.05] P = 0.02	-0.11[-0.25,0.03] P = 0.12	-0.20[-0.47,0.07] P = 0.14	-0.30[-0.56,-0.05] P = 0.02	-0.27[-0.47,-0.08] P = 0.006

MACE, major adverse cardiac events; LLL, late lumen loss; OR, odds ratio; MD, mean difference; CI, confidence interval; NA, not applicable; DEB, drug-eluting balloon; BMS, bare-metal stent; EPC, endothelial progenitor cell capturing; DES, drug-eluting stent; PES, paclitaxel-eluting stent; LES, limus-eluting stent; STEMI, ST-segment elevation myocardial infarction

## Discussion

This present meta-analysis involving 2281 patients showed that DEB with regular BMS was effective in treating *de novo* coronary disease. Treatment with DEB+BMS was superior to BMS therapy in angiographic and clinical follow-up because DEB+BMS versus BMS showed significantly reduced MACE incidence and LLL. Meanwhile, treatment with DEB+BMS was inferior to DES that it was associated with negative results in terms of MACE, LLL, TLR, and MLD. Furthermore, treatment with DEB+BMS was comparable to PES while tended to be inferior to second-generation DES based on a small sample size in subset analysis.

First-generation DES is restricted to prolonged dual antiplatelet therapy compared with BMS, with increased risk of late stent thrombosis due to incomplete endothelializsation of stent struts and an inflammatory response to the polymeric coating [35]. With the development of stent materials, platforms, and delivery systems, second-generation DES has significantly improved safety and efficacy outcomes compared with BMS and first-generation DES [5,6]. LEADERS FREE trial has shown a polymer-free umirolimus-coated stent was superior to a BMS in terms of the safety endpoints composite of cardiac death, myocardial infarction, and definite or probable stent thrombosis with a 1-month course of dual antiplatelet therapy in patients at high risk for bleeding [7]. While ZEUS trial has demonstrated a lower risk of 1-year MACE (all-cause death, nonfatal MI, and any target vessel revascularization) without increasing risk of bleeding or thrombosis in patients with contraindications to old DES [8]. In fact, DEB can provide an immediate and homogenous drug uptake without stent struts or polymers [36,37,38]. DEB used in combination with BMS has the potential to inhibit neointimal hyperplasia without delayed vascularization, preventing acute vessel wall recoil [37]. Due to the absence of polymers and short presence of drugs in the vessel wall, the duration of dual antiplatelet therapy can be shortened.

Previous meta-analysis demonstrated that DEB+BMS appeared to be superior to BMS for *de novo* coronary lesions, with no significant difference [13,14]. Recently, several RCTs have assessed DEB+BMS for the treatment of *de novo* CAD compared to BMS. In the present meta-analysis, DEB+BMS showed an overt advantage over BMS in terms of MACEs and LLL. Both first-generation (PES and SES) and second-generation (EES and ZES) DES were adapted. Previous studies [13,14] indicated that DEB with/without BMS is comparable to DES for treating *de novo* CAD. In this analysis, only studies applying DEB with regular BMS were eligible because trials that used DEB alone with bail-out BMS were different in design, which can cause pronounced heterogeneity.

The PES consisted of stainless steel of 97 to 147 mm that elutes paclitaxel from a durable polymer, which is associated with medial necrosis, positive remodeling, and excessive fibrin deposition. Previous studies showed that second-generation DES, such as EES or ZES, have improved outcomes with respect to MACEs, ST, and MI compared with the PES [39,40]. Furthermore, SES could reduce TLR rate compared with PES in short term follow up [40]. Interestingly, EES, ZES, and SES elute drugs in the limus family that share the same underlying pharmacological mechanism [40].

In this study, we compared DEB+BMS with PES exclusively, and found no statistically significant difference between both groups in terms of angiographic and clinical endpoints (Table 4). The PEPCAD III trial [24] reported that SES is superior to DEB+BMS in terms of clinical outcomes. This multicenter randomized trial enrolled 637 patients with *de-novo* coronary artery lesions from 24 sites. In a 9-month clinical follow-up, DEB+BMS was shown to be associated with a higher risk of MACEs (P<0.001) and TLR (P = 0.006). Obviously, this study obtained different results from reports comparing DEB+BMS and PES. In the current interventional practice, EES is the most common DES type used worldwide, in with the drug released from a biocompatible fluoro-copolymer coated onto a thinner cobalt—chromium stent [41]. Previous studies demonstrated that cobalt chromium EES is currently the most efficacious and safest device available for treating CAD [42,43]. The present meta-analysis only included three studies [25,26,27] comparing DEB+BMS with Xience V EES. Subset analysis showed that treatment with DEB+BMS tended to be inferior to Xience V EES in terms of MACEs (OR: 2.60, P = 0.08), LLL (MD: 0.31 mm, P = 0.06), TLR (OR: 3.45, P = 0.08), binary restenosis (OR: 5.23, P = 0.0006), and MLD (MD: -0.30 mm, P = 0.02). ZES is another second-generation DES, whose effectiveness has been confirmed [42]. A study conducted by Yoon et al. [30] enrolled patients with *de novo* non-small vessel CAD and compared DEB+BMS to Resolute Integrity ZES. With 180 patients randomly divided into DEB+BMS (n = 90) and ZES (n = 90) groups, the use of DEB+BMS was associated with disadvantageous outcomes compared to ZES regarding TLR (6.7% vs. 2.2%) and LLL (P<0.001). Nonetheless, studies comparing DEB+BMS to new generation DES are scarce. More studies are needed to provide additional insights into the relative DEB efficacy in comparison with new generation DES.

Various types of DEB were assessed in the present analysis. We compared SeQuent Please in the treatment of *de novo* CAD with DES. Overall, SeQuent Please+ BMS was comparable to DES in terms of angiographic and clinical outcomes, indicating that SeQuent Please might be a promising alternative to DES in *de novo* CAD. However, these results cannot be extrapolated to DEBs in general. In the DEB-AMI study [20], Dior-II balloons were shown to be inferior to Taxus Liberté, both in angiographic and clinical outcomes at follow up. Indeed, Dior utilizes a peculiar coating technology, where adherence of paclitaxel is mediated by a roughened surface of the balloon, whereas in the SeQuent Please paclitaxel is stuck to a water-soluble matrix. Compared with SeQuent Please, Dior may have failed to warrant sufficient bioavailability of paclitaxel at the lesion site [19,20,44].

When DEB is used in combination with regular BMS, geographical mismatch between DEB-dilated segment and stent coverage is considered a potential problem, which leads to higher rates of restenosis, especially focal restenosis [45]. In the OCTOPUS study [27], BMS was systematically post-dilated with a safety margin of 2.0–2.5 mm longer balloon to reduce geographic mismatch. However, treatment with DEB+BMS showed more late lumen loss (P = 0.034) and less net luminal gain (P = 0.064) compared to EES after 6-month angiographic follow up. A study by Zurakowski et al [31] selected DEB 3–4 mm longer than the BMS to avoid geographical mismatch, and MACEs, TLR, and LLL were comparable between the DEB+BMS and PES groups. Overall, geographical mismatch may be associated with negative outcomes of DEB+BMS, but do not constitute the main reason.

This meta-analysis presents a number of limitations that cannot be ignored. First, the sequence of devices (DEB first versus BMS first) may cause heterogeneity although a previous study found it doesn’t affect treatment outcomes [46]. Second, MACE definitions varied among RCTs, and we used the definitions reported in each study. As for LLL, most trials defined it with in-segment LLL, while distal main branch LLL was used in the DEBIUT trial [33], and in-stent LLL was reported in two studies [21,31]. Third, different types of DEBs have become an important source of heterogeneity. Therefore, sensitivity analysis was conducted by comparing SeQuent Please +BMS to DES. Fourth, the various comparators may be another source of heterogeneity. In the BMS group, EPC stents were applied in two studies [23,28] while in the DES group, both first- and second-generation DES were adapted. To mitigate heterogeneity, sensitivity analysis was conducted by excluding EPC stent, analyzing PES or EES exclusively. Nonetheless, information regarding comparison between DEB+BMS and second-generation DES was limited. Fifth, patients with STEMI or bifurcation lesions were involved in the study. In addition, follow-up duration in these trials was too short, especially for late ST, and long term follow-up RCTs are needed. Therefore, ST results should be interpreted carefully based on such small sample size studies.

## Conclusions

This meta-analysis showed that treatment with DEB appears to be effective in *de novo* coronary artery disease. In addition, DEB+ BMS was clearly superior to BMS and inferior to DES for the treatment of patients with *de novo* coronary artery disease. Hence, DES (especially new generation DES) should be recommended for patients with *de novo* CAD.

## Supporting information

S1 File PRISMA 2009 checklist. (DOCX)
